# Analysis of Electrolyte Abnormalities in Adolescents and Adults and Subsequent Diagnosis of an Eating Disorder

**DOI:** 10.1001/jamanetworkopen.2022.40809

**Published:** 2022-11-08

**Authors:** Gregory L. Hundemer, Anna Clarke, Ayub Akbari, Ann Bugeja, David Massicotte-Azarniouch, Greg Knoll, Daniel T. Myran, Peter Tanuseputro, Manish M. Sood

**Affiliations:** 1 Division of Nephrology, Department of Medicine, University of Ottawa, Ottawa, Ontario, Canada; 2 Clinical Epidemiology Program, Ottawa Hospital Research Institute, University of Ottawa, Ottawa, Ontario, Canada; 3 ICES (formerly Institute for Clinical Evaluative Sciences), Ottawa, Ontario, Canada; 4 Department of Family Medicine, University of Ottawa, Ottawa, Ontario, Canada; 5 Division of Palliative Care, Department of Medicine, University of Ottawa, Ottawa, Ontario, Canada

## Abstract

**Importance:**

Eating disorders lead to increased mortality and reduced quality of life. While the acute presentations of eating disorders frequently involve electrolyte abnormalities, it remains unknown whether electrolyte abnormalities may precede the future diagnosis of an eating disorder.

**Objective:**

To determine whether outpatient electrolyte abnormalities are associated with the future diagnosis of an eating disorder.

**Design, Setting, and Participants:**

This population-level case-control study used provincial administrative health data for residents of Ontario, Canada aged 13 years or older from 2008 to 2020. Individuals without an eating disorder (controls) were matched 4:1 to individuals diagnosed with an incident eating disorder (cases) based on age and sex. Both groups had outpatient electrolyte measurements between 3 years and 30 days prior to index. Index was defined as the date of an eating disorder diagnosis in any inpatient or outpatient clinical setting for cases. Controls were assigned a pseudo-index date according to the distribution of index dates in the case population. Individuals with any prior eating disorder diagnosis were excluded. The data analyzed was from January 1, 2008, through June 30, 2020.

**Exposures:**

Any electrolyte abnormality, defined as abnormal test results for a composite of hypokalemia, hyperkalemia, hyponatremia, hypernatremia, hypomagnesemia, hypophosphatemia, metabolic acidosis, or metabolic alkalosis.

**Outcomes and Measures:**

Eating disorder diagnosis including anorexia nervosa, bulimia nervosa, and eating disorder not otherwise specified.

**Results:**

A total 6970 eligible Ontario residents with an eating disorder (mean [SD] age, 28 (19) years; 6075 [87.2%] female, 895 [12.8%] male) were matched with 27 878 age- and sex-matched residents without an eating disorder diagnosis (mean [SD] age, 28 [19] years; 24 300 [87.2%] female, 3578 [12.8%] male). Overall, 18.4% of individuals with an eating disorder had a preceding electrolyte abnormality vs 7.5% of individuals without an eating disorder (adjusted odds ratio [aOR], 2.12; [95% CI, 1.86-2.41]). The median (IQR) time from the earliest electrolyte abnormality to eating disorder diagnosis was 386 (157-716) days. Specific electrolyte abnormalities associated with a higher risk of an eating disorder were: hypokalemia (aOR, 1.98; 95% CI, 1.70-2.32), hyperkalemia (aOR, 1.97; 95% CI, 1.48-2.62), hyponatremia (aOR, 5.26; 95% CI, 3.32-8.31), hypernatremia (aOR, 3.09; 95% CI, 1.01-9.51), hypophosphatemia (aOR, 2.83; 95% CI, 1.82-4.40), and metabolic alkalosis (aOR, 2.60; 95% CI, 1.63-4.15).

**Conclusions and Relevance:**

In this case-control study, individuals with an eating disorder were associated with a preceding outpatient electrolyte abnormality compared with matched controls. Otherwise unexplained electrolyte abnormalities may serve to identify individuals who may benefit from screening for an underlying eating disorder.

## Key Points

**Question:**

Are electrolyte abnormalities in outpatient lab work associated with an eating disorder diagnosis in the future?

**Findings:**

In this population-based case-control study of individuals with and without an incident eating disorder, an outpatient electrolyte abnormality 3 years to 30 days prior to diagnosis was associated with 2.1-fold higher odds for the subsequent diagnosis of an eating disorder, a significant difference.

**Meaning:**

These results suggest that otherwise unexplained outpatient electrolyte abnormalities may serve to identify individuals who should be screened for an underlying eating disorder.

## Teaser

This case-control study of residents in Ontario, Canada over age 13 years examines the association of eating disorders with abnormal results in electrolyte tests.

## Introduction

Eating disorders, such as anorexia nervosa and bulimia nervosa, are defined as disturbances in eating behaviors that compromise health and impair normal functioning.^1^ The lifetime prevalence of an eating disorder is approximately 2% and is more common among women and adolescents.^2^ Eating disorders are strongly linked to increased morbidity and mortality along with reduced quality of life.^3,4,5^ These conditions present many challenges to both patients and clinicians.^6,7,8^ Such challenges include an overall lack of adequate treatment options along with patient-centered factors such as denial, lack of self-awareness, social stigma, and shame surrounding the diagnosis that may limit forthcoming discussion and delay both diagnosis and treatment.^9^ Further tools to aid in prompting or confirming suspicion into an underlying eating disorder may improve screening and promote early detection.

The acute presentations of eating disorders may involve marked electrolyte abnormalities. These include imbalances in potassium, sodium, magnesium, and phosphate along with acid-base disturbances.^10,11,12,13,14^ In terms of prevalence, a study of 1026 consecutive adults admitted for an eating disorder found that at the time of admission, 25.8% had hypokalemia, 14.0% had hyponatremia, and 16.6% had metabolic alkalosis.^15^ The prevalence of electrolyte abnormalities in the outpatient setting among individuals with eating disorders is not well understood. These electrolyte abnormalities are often severe and predispose to such adverse and potentially fatal health consequences as cardiac arrhythmias, muscle weakness, and mental status changes. The pathogenesis underlying these electrolyte abnormalities can be explained by a number of etiologies relating to behaviors directly tied to the eating disorder including purging by vomiting, diuretic or laxative abuse, inadequate intake, and dehydration. However, it remains largely unknown whether early electrolyte abnormalities, prior to the acute presentation of an eating disorder, are associated with the future diagnosis of an eating disorder.

While several screening tools to identify individuals with eating disorders have been developed, such as the SCOFF questionnaire (Sick, Control, One stone, Fat, Food),^16,17^ they require an index of suspicion prior to screening. Incidentally discovered electrolyte abnormalities, if associated with the subsequent diagnosis of an eating disorder, may help raise suspicion for clinicians to identify patients in whom more intensive screening into a potential eating disorder may be warranted. Herein, we conducted a large population-level case-control study of individuals in Ontario, Canada to evaluate the association between outpatient electrolyte abnormalities and the future diagnosis of an eating disorder.

## Methods

### Study Design and Setting

We conducted a population-level, case-control study of individuals aged 13 years or older in Ontario, Canada from January 1, 2008, through June 30, 2020. Ontario is Canada’s largest province with approximately 15 million residents.^18^ We used linked data sets held at ICES (formerly the Institute for Clinical Evaluative Sciences). ICES is an independent, nonprofit research institute whose legal status under Ontario’s health information privacy law allows it to collect and analyze health care and demographic data, without consent, for health system evaluation and improvement. ICES captures data on all Ontario residents who undergo a health care encounter including health care visits, laboratory tests, hospitalizations, and vital statistics. The use of data in this project was authorized under section 45 of Ontario’s Personal Health Information Protection Act, which does not require review by a research ethics board or informed consent. The reporting of this study follows the Strengthening the Reporting of Observational Studies in Epidemiology (STROBE) reporting guidelines for case-control studies (eTable 1 in the Supplement).

### Data Sources

We ascertained patient characteristics and outcome data from de-identified, linked databases housed at ICES. Demographic and vital status information was obtained from the Ontario Registered Persons Database. Diagnostic and procedural information from all hospitalizations were determined using the Canadian Institute for Health Information Discharge Abstract Database. Diagnostic information from ambulatory, emergency department, and day surgery visits was determined using the National Ambulatory Care Reporting System. Information was also obtained from the Ontario Health Insurance Plan (OHIP) database, which contains all health claims for inpatient and outpatient physician services. Definitions for patient characteristics and clinical variables can be found in eTable 2 in the Supplement. Laboratory information is contained in the Ontario Laboratory Information System, which captures laboratory tests for individuals in Ontario. These data sets were linked using unique encoded identifiers and analyzed at ICES. The databases were complete for all other variables used except for rural residence and neighborhood income quintile, which were missing for fewer than 0.5% of individuals.

### Case and Control Definitions

**Figure 1.**
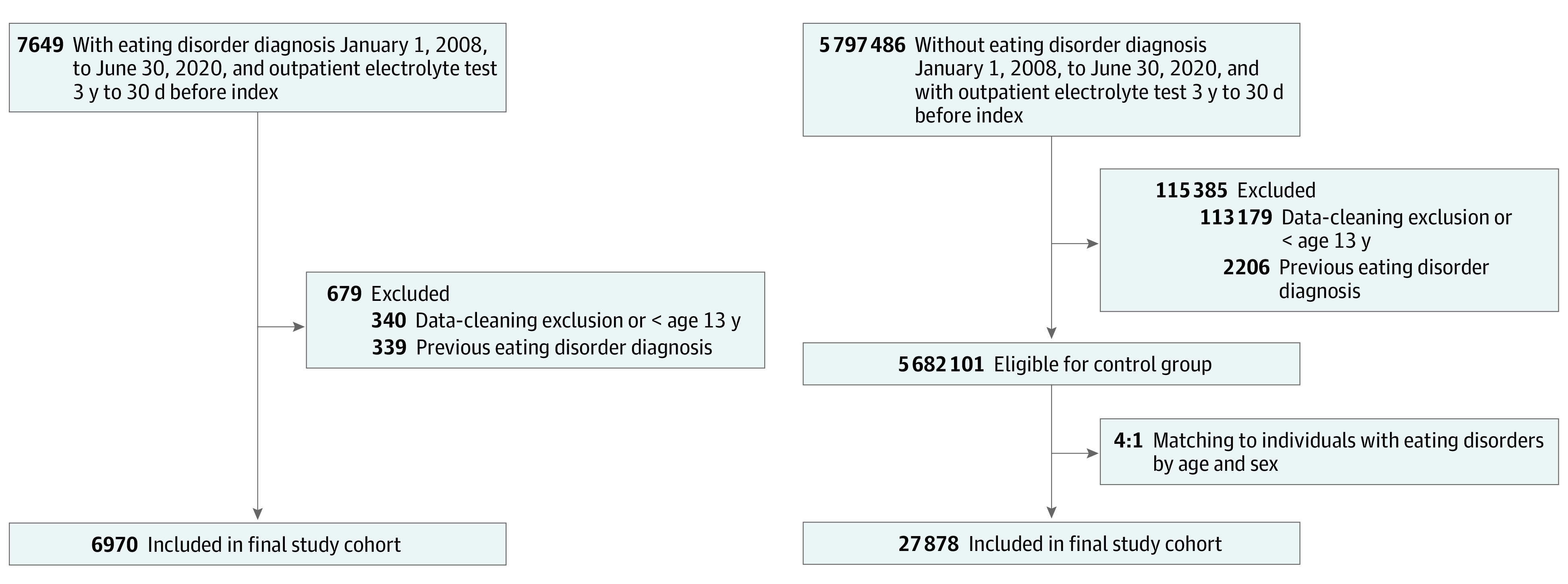
Flowchart for Study Assembly

Cases and controls were defined as individuals in Ontario who were aged 13 years or older and had at least 1 outpatient electrolyte test between 3 years and 30 days prior to index (Figure 1). Cases were individuals diagnosed with an incident eating disorder between January 1, 2008, and June 30, 2020. The index date for cases was the first diagnosis date of an eating disorder within the accrual period. Controls were randomly assigned a pseudoindex date according to the distribution of index dates in the case population.^19^ Eating disorder diagnoses were based upon diagnostic codes for anorexia nervosa, bulimia nervosa, and eating disorder not otherwise specified. Eating disorder diagnoses were captured in all clinical settings (ie, inpatient, outpatient, and emergency department encounters). Individuals were excluded if they had any prior history of an eating disorder diagnosis before the study period. Controls were matched 4:1 to cases by age (exact) and sex.

### Exposure

The primary study exposure was at least 1 outpatient electrolyte abnormality, defined by any of the following test results: hypokalemia (serum potassium level 3.5 mmol/L or lower), hyperkalemia (serum potassium, 5.5 mmol/L or higher), hyponatremia (serum sodium, 130 mmol/L or lower), hypernatremia (serum sodium, 150 mmol/L or higher), hypomagnesemia (serum magnesium, 0.50 mmol/L or lower), hypophosphatemia (serum phosphate, 0.80 mmol/L or lower), metabolic acidosis (serum bicarbonate, 22 mmol/L or lower), or metabolic alkalosis (serum bicarbonate, 30 mmol/L or higher). We included only outpatient electrolyte measurements so as to avoid common acute medical issues that can result in electrolyte disturbances. The exposure was labeled categorically in regard to whether they developed an electrolyte abnormality within the study window or not. Only electrolyte measurements between 3 years and 30 days prior to the index date were included. Electrolyte abnormalities within 30 days prior to index were excluded as they may be associated with the acute presentation of an eating disorder diagnosis. We further examined each individual electrolyte abnormality separately. Individuals who developed more than 1 type of electrolyte abnormality (eg, hypokalemia and metabolic alkalosis) within the study period were labeled accordingly. Moreover, in an additional analysis we incorporated a more severe definition for each electrolyte abnormality as follows: hypokalemia (serum potassium levels of 3.0 mmol/L or below), hyperkalemia (serum potassium, 6.0 mmol/L or higher), hyponatremia (serum sodium, 128 mmol/L or lower), hypernatremia (serum sodium, 155 mmol/L or higher), hypomagnesemia (serum magnesium, 0.40 mmol/L or lower), and hypophosphatemia (serum phosphate, 0.60 mmol/L or lower).

### Outcomes

The outcome of interest was the diagnosis of an eating disorder based upon diagnostic codes for anorexia nervosa, bulimia nervosa, or eating disorder not otherwise specified. These validated codes have previously been used to identify eating disorders in Ontario, Canada.^20,21^ Eating disorder diagnoses were captured in all clinical settings (ie, inpatient, outpatient, and emergency department encounters).

### Statistical Analysis

We compared characteristics between the cases (individual diagnosed with an eating disorder) and controls (age- and sex-matched individuals without an eating disorder diagnosis). Continuous variables were reported as mean (with standard deviation [SD]) while categorical variables were reported as overall number (with percentages). We then calculated the percentage of cases and controls with electrolyte abnormalities detected between 3 years and 30 days prior to the index date. We used both univariable and multivariable logistic regression models to measure both crude and adjusted odds ratios (aOR) along with 95% CIs in the association between any electrolyte abnormality and an eating disorder diagnosis. Our models adjusted for the following covariates selected a priori: age, sex, year of index, neighborhood income quintile, rural residence, number of hospitalizations in the prior 2 years, prior health care visit for mental health, prior outpatient psychiatry visit, anxiety, asthma, chronic kidney disease, chronic obstructive pulmonary disease, congestive heart failure, depression, diabetes mellitus, inflammatory bowel disease, liver disease, myocardial infarction, personality disorder, and substance abuse. Race and ethnicity data is not available in ICES and therefore was not included in the study. We repeated our models using the more severe definitions for electrolyte abnormalities described above. We conducted all analyses using SAS Enterprise Guide version 7.1 (SAS Institute Inc). Confidence intervals that did not overlap with 1.0 and 2-sided *P* values <.05 were treated as statistically significant.

## Results

### Patient Characteristics

The study included 6970 eligible Ontario residents diagnosed with an eating disorder (cases; mean [SD] age, 28 [19] years; 6075 [87.2%] female; 895 [12.8%] male) and 27 878 age- and sex-matched eligible Ontario residents without an eating disorder diagnosis (controls; mean [SD] age, 28 [19] years; 24 300 [87.2%] female, 3578 [12.8%] male) (Figure 1) (Table). Individuals with an eating disorder had higher rates of hospitalization (hospitalizations within 2 years: 1864 of 6970 [26.7%] vs 3136 of 27 787 [11.2%]) along with a higher prevalence of psychiatric comorbidities including anxiety (1372 [19.7%] vs 1320 [4.7%]), depression (1346 [19.3%] vs 951 [3.4%]), personality disorder (299 [4.3%] vs 108 [0.4%]), and substance abuse (567 [8.1%] vs 521 [1.9%]).

**Table.** Characteristics of Individuals With (Cases) and Without (Controls) an Eating Disorder Diagnosis

Characteristic	Individuals, No. (%)	
	Cases (n = 6970)	Controls (n = 27 878)
Age, mean (SD), y	28 (19)	28 (19)
Sex		
Female	6075 (87.2)	24 300 (87.2)
Male	895 (12.8)	3578 (12.8)
Index year		
2008	64 (1.0)	9 (<0.1)
2009	213 (3.1)	149 (0.5)
2010	320 (4.6)	517 (1.9)
2011	438 (6.3)	1016 (3.6)
2012	611 (8.8)	1830 (6.6)
2013	638 (9.2)	2031 (7.3)
2014	686 (9.8)	2382 (8.5)
2015	667 (9.6)	2521 (9.0)
2016	681 (9.8)	2894 (10.4)
2017	779 (11.2)	3606 (12.9)
2018	771 (11.1)	3928 (14.1)
2019	758 (10.9)	4417 (15.8)
2020	344 (4.9)	2578 (9.2)
Neighborhood income, quintile^a^		
1st	1401 (20.1)	5305 (19.0)
2nd	1255 (18.0)	5344 (19.2)
3rd	1294 (18.6)	5725 (20.5)
4th	1344 (19.3)	5694 (20.4)
5th	1622 (23.3)	5722 (20.5)
Rural^b^	663 (9.5)	2693 (9.7)
Health care exposure		
Hospitalizations within prior 2 y		
0	5106 (73.3)	24 742 (88.8)
1	1036 (14.9)	2397 (8.6)
2	383 (5.5)	467 (1.7)
≥3	445 (6.4)	272 (1.0)
Prior health care visit for mental health	2830 (40.6)	2836 (10.2)
Prior outpatient psychiatry visit	3691 (53.0)	3656 (13.1)
Comorbidities^c^		
Anxiety	1372 (19.7)	1320 (4.7)
Asthma	1205 (17.3)	3754 (13.5)
Chronic kidney disease	199 (2.9)	401 (1.4)
Chronic obstructive pulmonary disease	319 (4.6)	636 (2.3)
Congestive heart failure	218 (3.1)	387 (1.4)
Depression	1346 (19.3)	951 (3.4)
Diabetes	632 (9.1)	2042 (7.3)
Inflammatory bowel disease	125 (1.8)	302 (1.1)
Liver disease	294 (4.2)	653 (2.3)
Myocardial infarction	36 (0.5)	105 (0.4)
Personality disorder	299 (4.3)	108 (0.4)
Substance abuse	567 (8.1)	521 (1.9)

^a^ Neighborhood income quintile missing in 142 individuals (0.4% of study population).

^b^ Rural defined as residing in a location with population <10,000, missing in 104 individuals (0.3% of study population).

^c^ Comorbidities were ascertained in the 5 years prior to index.

### Prevalence of Electrolyte Abnormalities

**Figure 2.**
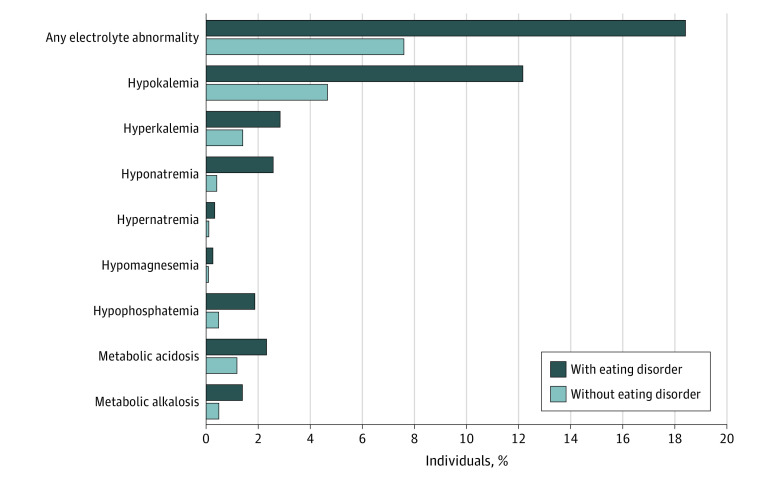
Electrolyte Abnormalities Among Individuals With and Without Eating Disorders Electrolyte abnormalities were defined as follows: hypokalemia (serum potassium ≤3.5 mmol/L), hyperkalemia (serum potassium ≥5.5 mmol/L), hyponatremia (serum sodium ≤130 mmol/L), hypernatremia (serum sodium ≥150 mmol/L), hypomagnesemia (serum magnesium ≤0.50 mmol/L), hypophosphatemia (serum phosphate ≤0.80 mmol/L), metabolic acidosis (serum bicarbonate ≤22 mmol/L), or metabolic alkalosis (serum bicarbonate ≥30 mmol/L).

By design, all electrolyte abnormalities preceded the index date (which for cases was the date the incident eating disorder was diagnosed) by a minimum of 30 days. However, the median (IQR) time from the earliest electrolyte abnormality (within the preceding 3-year to 30-day study window) to index was much longer: 386 (157-716) days for cases and 497 (239-803) days for controls. Individuals with an eating disorder were more likely to have an electrolyte abnormality compared with individuals without an eating disorder (1281 of 6970 [18.4%] vs 2104 of 27 878 [7.5%]; *P* < .001) (Figure 2). Moreover, each individual electrolyte abnormality was more common in individuals diagnosed with an eating disorder compared with those who were not: hypokalemia (845 of 6970 [12.1%] vs 1288 of 27 878 [4.6%]), hyperkalemia (195 of 6970 [2.8%] vs 376 of 27 878 [1.3%]), hyponatremia (176 of 6970 [2.5%] vs 100 of 27 878 [0.4%]), hypernatremia (20 of 6970 [0.3%] vs 19 of 27 878 [0.1%]), hypomagnesemia (17 of 6970 [0.2%] vs 15 of 27 878 [0.1%]), hypophosphatemia (129 of 6970 [1.9%] vs 122 of 27 878 [0.4%]), metabolic acidosis (160 of 6970 [2.3%] vs 321 of 27 878 [1.2%]), and metabolic alkalosis (95 of 6970 [1.4%] vs 119 of 27 878 [0.4%]) (*P* < .001). The median (IQR) number of electrolyte measurements within the study window was 2 (1-3) and 1 (1-2) for cases and controls, respectively.

### Association Between Electrolyte Abnormalities and the Subsequent Diagnosis of an Eating Disorder

**Figure 3.**
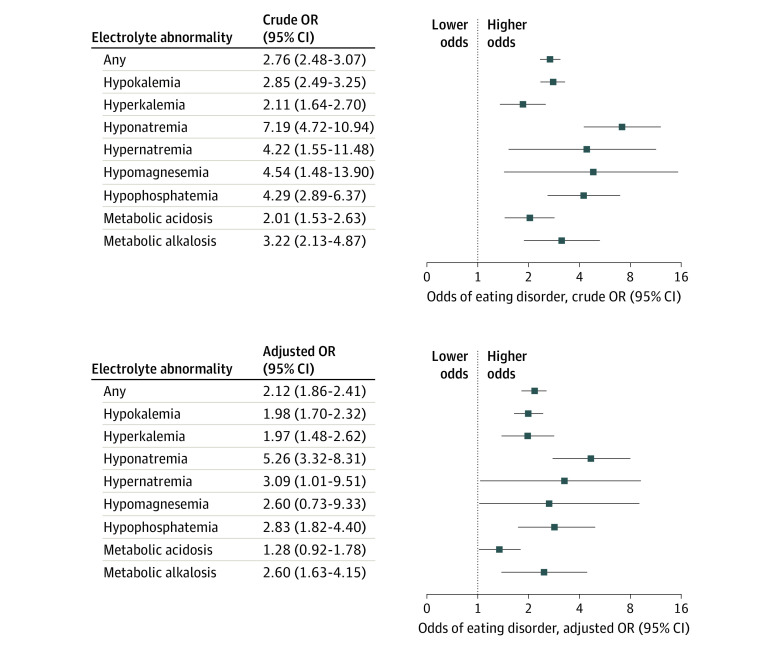
Crude and Adjusted Odds Ratios for the Association Between Electrolyte Abnormalities and Subsequent Eating Disorder Diagnosis OR indicates odds ratios. Multivariable models were adjusted for the following covariates: age, sex, year of index, income quintile, rural residence, number of hospitalizations in the prior 2 years, prior health care visit for mental health, prior outpatient psychiatry visit, anxiety, asthma, chronic kidney disease, chronic obstructive pulmonary disease, congestive heart failure, depression, diabetes, inflammatory bowel disease, liver disease, myocardial infarction, personality disorder, and substance abuse.

Any outpatient electrolyte abnormality was associated with an over 2-fold higher odds of a subsequent eating disorder diagnosis (aOR, 2.12; 95% CI, 1.86-2.41) (Figure 3). The following individual electrolyte abnormalities were associated with a higher risk of a subsequent eating disorder diagnosis: hypokalemia (aOR, 1.98; 95% CI, 1.70-2.32), hyperkalemia (aOR, 1.97; 95% CI, 1.48-2.62), hyponatremia (aOR, 5.26; 95% CI, 3.32-8.31), hypernatremia (aOR. 3.09; 95% CI, 1.01-9.51), hypophosphatemia (aOR, 2.83; 95% CI, 1.82-4.40), and metabolic alkalosis (aOR, 2.60; 95% CI, 1.63-4.15). There was no significant difference in the risk of a subsequent eating disorder diagnosis with hypomagnesemia (aOR, 2.60; 95% CI, 0.73-9.33) or metabolic acidosis (aOR, 1.28; 95% CI, 0.92-1.78).

The association between electrolyte abnormalities and the subsequent diagnosis of an eating disorder was also analyzed using more severe cutoffs (hypokalemia, serum potassium levels of 3.0 mmol/L or lower; hyperkalemia, serum potassium 6.0 mmol/L or higher; hyponatremia, serum sodium 128 mmol/L or lower; hypernatremia, serum sodium 155 mmol/L or higher; hypomagnesemia, serum magnesium 0.40 mmol/L or lower; hypophosphatemia, serum phosphate 0.60 mmol/L or lower) (eFigure in the Supplement). Severe hypokalemia (aOR, 7.20; 95% CI, 4.22-12.26), severe hyperkalemia (aOR, 2.17; 95% CI, 1.27-3.72), and severe hyponatremia (aOR, 5.30; 95% CI, 2.82-9.95) were associated with an increased risk of a subsequent eating disorder diagnosis. There was no significant difference in the risk of a subsequent eating disorder diagnosis with severe hypophosphatemia (aOR, 1.84; 95% CI, 0.66-5.13). Severe hypernatremia and severe hypomagnesemia were not examined in our models as there were 10 individuals or fewer in the overall study population with these electrolyte abnormalities.

## Discussion

Diagnosing an eating disorder is often challenging and, as such, routinely and readily available tests that may prompt health care providers to screen for these conditions may be beneficial. In this large population-based case-control study of individuals ages 13 years or older in Ontario, Canada without a prior eating disorder diagnosis, we found that an outpatient electrolyte abnormality was associated with an over 2-fold higher odds of a subsequent eating disorder diagnosis. The median time from the earliest electrolyte abnormality to eating disorder diagnosis exceeded one year. Hypokalemia was the most common electrolyte abnormality occurring in 12.1% of individual diagnosed with an eating disorder compared with 4.6% in matched controls. Additional electrolyte abnormalities such as hyponatremia, hypernatremia, hypophosphatemia, and metabolic alkalosis, although less frequent in their occurrence, were independently associated with an even higher odds of an eating disorder diagnosis. Our findings were consistent when we examined more severe electrolyte abnormalities including severe hypokalemia (serum potassium levels of 3.0 mmol/L or lower) and severe hyponatremia (serum sodium, 128 mmol/L or lower), which were associated with over 7-fold and 5-fold higher odds for the diagnosis of an eating disorder, respectively.

Our results expand upon prior studies examining the coexistence of eating disorders and electrolyte abnormalities, which are often severe.^10,11,12,13,14^ For example, a US study^15^ of 1026 consecutive individuals admitted to an eating recovery center found that electrolyte abnormalities were common at the time of admission as 25.8%, 14.0%, 5.7%, and 16.6% had hypokalemia, hyponatremia, hypophosphatemia, and metabolic alkalosis, respectively. Whereas these prior studies have focused on the presence of electrolyte abnormalities during the acute presentation of an eating disorder or during the refeeding process, the novelty in the current study was that these electrolyte abnormalities were present well before the eating disorder diagnosis was made and occur in the ambulatory (ie, outpatient) setting. In our study, we included only outpatient electrolyte measurements taken 30 days or more prior to the diagnosis of an eating disorder with the majority of electrolyte abnormalities preceding the eating disorder diagnosis by over 1 year. In the population of Ontario, Canada, we found the most common electrolyte abnormality to precede an eating disorder diagnosis to be hypokalemia though there was also a significant association with a number of other electrolyte abnormalities including hyperkalemia, hyponatremia, hypernatremia, hypophosphatemia, and metabolic alkalosis.

These results may help clinicians identify patients who may benefit from specific screening for an underlying eating disorder or help to confirm a suspected diagnosis. Several screening tools of varying complexity have been developed which are validated and accurate in identifying individuals with a potential eating disorder. These include the SCOFF questionnaire,^16,17^ the Eating Disorder Screen for Primary Care,^22^ the Eating Attitudes Test,^23^ and the Primary Care Evaluation of Mental Disorders Patient Health Questionnaire.^24^ However, widespread routine screening for eating disorders is neither practical nor cost-effective and as such is not recommended by the US Preventive Services Task Force.^2^ As these study results demonstrate, an incidental electrolyte abnormality may aid clinicians in identifying candidates at high risk for an underlying eating disorder whom they should consider screening. In turn, this may allow for earlier diagnosis of, and intervention for, eating disorders and potentially improve outcomes.

### Strengths and Limitations

We would like to highlight several unique strengths with our current study. Rather than a single-center or multi-center design, our study was population-based and covered a wide, heterogeneous landscape across Canada’s largest province of Ontario. Also, we only included outpatient electrolyte measurements so as to avoid common acute medical issues that can result in electrolyte disturbances. We also required that the electrolyte measurements be taken beyond 30 days prior to the eating disorder diagnosis. In fact, we found that the median time from the earliest electrolyte abnormality within the study window to the diagnosis of an eating disorder was greater than 1 year. This aligned with our goal of determining whether electrolyte disturbances identified as part of outpatient care may forecast future eating disorder diagnoses.

This study had several limitations. Our results must be interpreted within the context of the study design. First, there was an imbalance between cases (ultimately diagnosed with an eating disorder) and controls (not diagnosed with an eating disorder) in regard to psychiatric comorbidities such as anxiety, depression, personality disorder, and substance abuse. This is not surprising as there is a large evidence base of the frequent overlap between eating disorders and comorbid psychiatric illness.^1,25,26,27,28^ However, the strong association between electrolyte abnormalities and subsequent eating disorder diagnosis persisted despite adjusting for these factors within our analyses. Second, comprehensive medication data in Ontario was only available for individuals ages 65 years or older, which represented only a minority of our study population. Therefore, we were unable to assess for prescription laxative, diuretic, or psychotropic medication (eg, selective serotonin reuptake inhibitors, lithium) use as a contributing factor to the electrolyte abnormalities. Third, we were unable to identify what type of clinician (eg, physician [primary care vs subspecialty], dietician, etc.) made the eating disorder diagnosis. In countries such as the US and Canada, there may be a reluctance to use eating disorder diagnosis codes for fear of lack of reimbursement, which may lead to a discrepancy in which types of clinicians employ these codes. This may have resulted in some individuals being labeled as controls despite potentially being treated for eating disorders and biasing the results toward the null. Finally, as this study was performed using administrative health data, we cannot comment on the reason that electrolyte measurements were performed on an individual patient basis. Eating disorders often predispose to concurrent illnesses (eg, volume depletion, infections) that may have prompted the laboratory testing and may explain why individuals with an eating disorder had a higher median number of electrolyte measurements. Furthermore, we cannot rule out whether a clinician may have had an index of suspicion for an eating disorder which was the reason behind measuring electrolyte levels in certain instances.

## Conclusions

In this population-based case-control study, we found that outpatient electrolyte abnormalities may serve as a precursor to future eating disorder diagnoses. Electrolyte abnormalities commonly occurred well ahead of the time at which an eating disorder diagnosis was made. Hypokalemia was the most common electrolyte abnormality identified while hyponatremia, hypernatremia, hypophosphatemia, and metabolic alkalosis were the most specific for a subsequent eating disorder diagnosis. These results suggest that incidentally discovered electrolyte abnormalities may serve to identify individuals who may benefit from targeted screening for an underlying eating disorder. Ultimately, this may allow for more timely diagnosis and intervention to mitigate the negative impact that eating disorders have on patient morbidity, mortality, and quality of life.

## Notes

Supplement. **eTable 1.** STROBE Checklist of Items That Should Be Included in Reports of Case-Control Studies **eTable 2.** Databases and Coding Definitions for Inclusion and Exclusion Criteria, Outcomes, and Patient Characteristics **eFigure.** Crude and Adjusted Odds Ratios for the Association Between Severe Electrolyte Abnormalities and Subsequent Eating Disorder Diagnosis
