# Supplementary material for: Analysis of Electrolyte Abnormalities in Adolescents and Adults and Subsequent Diagnosis of an Eating Disorder
Source: JAMA Netw Open. 2022 Nov 8;5(11):e2240809. doi: 10.1001/jamanetworkopen.2022.40809 (PMC9644262; doi:10.1001/jamanetworkopen.2022.40809)
Supplement: Supplement. — eTable 1. STROBE Checklist of Items That Should Be Included in Reports of Case-Control Studies eTable 2. Databases and Coding Definitions for Inclusion and Exclusion Criteria, Outcomes, and Patient Characteristics eFigure. Crude and Adjusted Odds Ratios for the Association Between Severe Electrolyte Abnormalities and Subsequent Eating Disorder Diagnosis [file jamanetwopen-e2240809-s001.pdf]

Supplemental Online Content

Hundemer GL, Clarke A, Akbari A, et al. Analysis of electrolyte abnormalities in adolescents and adults and subsequent diagnosis of an eating disorder. *JAMA Netw Open.* 2022;5(11):e2240809. doi:10.1001/jamanetworkopen.2022.40809

eTable 1. STROBE Checklist of Items That Should Be Included in Reports of Case-Control Studies

eTable 2. Databases and Coding Definitions for Inclusion and Exclusion Criteria, Outcomes, and Patient Characteristics

eFigure. Crude and Adjusted Odds Ratios for the Association Between Severe Electrolyte Abnormalities and Subsequent Eating Disorder Diagnosis

This supplemental material has been provided by the authors to give readers additional information about their work.

© 2022 Hundemer GL et al. *JAMA Network Open.*

eTable 1: STROBE checklist of items that should be included in reports of case-control studies.

	Item No	STROBE items	Reported
**Title and abstract**	1	(a) Indicate the study's design with a commonly used term in the title or the abstract.<br><br>(b) Provide in the abstract an informative and balanced summary of what was done and what was found.	Abstract
**Introduction**			
Background/ rationale	2	Explain the scientific background and rationale for the investigation being reported.	Introduction
Objectives	3	State specific objectives, including any prespecified hypotheses.	Introduction
**Methods**			
Study design	4	Present key elements of study design early in the paper.	Methods
Setting	5	Describe the setting, locations, and relevant dates, including periods of recruitment, exposure, follow-up, and data collection.	Methods
Participants	6	(a) Give the eligibility criteria, and the sources and methods of case ascertainment and control selection. Give the rationale for the choice of cases and controls.<br><br>(b) For matched studies, give matching criteria and the number of controls per case.	Methods
Variables	7	Clearly define all outcomes, exposures, predictors, potential confounders, and effect modifiers. Give diagnostic criteria, if applicable.	Methods
Data sources/ measurement	8	For each variable of interest, give sources of data and details of methods of assessment (measurement). Describe comparability of assessment methods if there is more than one group.	Methods, eTable 2
Bias	9	Describe any efforts to address potential sources of bias.	Methods

© 2022 Hundemer GL et al. *JAMA Network Open.*

Study size	10	Explain how the study size was arrived at.	Methods, Figure 1
Quantitative variables	11	Explain how quantitative variables were handled in the analyses. If applicable, describe which groupings were chosen and why.	Methods
Statistical methods	12	(a) Describe all statistical methods, including those used to control for confounding.<br>(b) Describe any methods used to examine subgroups and interactions.<br>(c) Explain how missing data were addressed.<br>(d) If applicable, explain how matching of cases and controls was addressed.<br>(e) Describe any sensitivity analyses.	Methods
**Results**			
Participants	13	(a) Report numbers of individuals at each stage of study--e.g., numbers potentially eligible, examined for eligibility, confirmed eligible, included in the study, completing follow-up, and analyzed.<br>(b) Give reasons for non-participation at each stage.<br>(c) Consider use of a flow diagram.	Results, Figure 1
Descriptive data	14	(a) Give characteristics of study participants (e.g., demographic, clinical, social) and information on exposures and potential confounders.<br>(b) Indicate number of participants with missing data for each variable of interest.	Results, Table 1
Outcome data	15	Report numbers in each exposure category, or summary measures of exposure.	Results, Figure 2
Main results	16	(a) Give unadjusted estimates and, if applicable, confounder-adjusted estimates and their precision (e.g., 95% confidence interval). Make clear which confounders were adjusted for and why they were included.<br>(b) Report category boundaries when continuous variables were categorized.<br>(c) If relevant, consider translating estimates of relative risk into absolute risk for a meaningful time period.	Results, Figure 3

© 2022 Hundemer GL et al. *JAMA Network Open.*

Other analyses	17	Report other analyses done (e.g., analyses of subgroups and interactions, and sensitivity analyses).	Results, eFigure 1
**Discussion**			
Key results	18	Summarize key results with reference to study objectives.	Discussion
Limitations	19	Discuss limitations of the study, taking into account sources of potential bias or imprecision. Discuss both direction and magnitude of any potential bias.	Discussion
Interpretation	20	Give a cautious overall interpretation of results considering objectives, limitations, multiplicity of analyses, results from similar studies, and other relevant evidence.	Discussion
Generalizability	21	Discuss the generalizability (external validity) of the study results.	Discussion
**Other information**			
Funding	22	Give the source of funding and the role of the funders for the present study and, if applicable, for the original study on which the present article is based.	Funding

© 2022 Hundemer GL et al. *JAMA Network Open.*

eTable 2: Databases and coding definitions for inclusion and exclusion criteria, outcomes, and patient characteristics.

Characteristic/Condition	Database	Codes
**Inclusion Criteria**		
Cases		
All individuals with an eating disorder diagnosis between 01JAN2008 and 30JUN2020<br>*Earliest diagnosis date listed as index date	DAD<br>NACRS	ICD10: F50.0, F50.1, F50.2, F50.3, F50.8, F50.9
Outpatient electrolyte test between 3 years and 30 days prior to index	OLIS<br>DAD<br>SDS<br>NACRS	OLIS observation codes:<br>2823-3<br>39789-3<br>6298-4<br>14627-4<br>1959-6<br>1960-4<br>1961-2<br>1962-0<br>1963-8<br>2951-2<br>2601-3<br>14879-1<br>24519-1
Controls		
All individuals eligible in RPDB at some point during accrual period (01JAN2008 through 30JUN2020)	RPDB	
Exclude those meeting case definition (eating disorder during accrual)	DAD<br>NACRS	ICD10: F50.0, F50.1, F50.2, F50.3, F50.8, F50.9
Assign pseudo-index date		

© 2022 Hundemer GL et al. *JAMA Network Open.*

Outpatient electrolyte test between 3 years and 30 days prior to index	OLIS<br>DAD<br>SDS<br>NACRS	OLIS observation codes:<br>2823-3<br>39789-3<br>6298-4<br>14627-4<br>1959-6<br>1960-4<br>1961-2<br>1962-0<br>1963-8<br>2951-2<br>2601-3<br>14879-1<br>24519-1
**Exclusion Criteria**		
Data cleaning<br>a. Missing or invalid IKN<br>b. Missing or invalid age (>105 years)<br>c. Missing or invalid sex<br>d. Death on or before the index date<br>e. Non-Ontario resident	RPDB	
Age <13 years at index date	RPDB	
Exclude those with a previous eating disorder diagnosis	DAD<br>NACRS	ICD10: F50.0, F50.1, F50.2, F50.3, F50.8, F50.9
**Eating Disorders**		
Anorexia nervosa	DAD<br>NACRS	ICD10: F50.0, F50.1
Bulimia nervosa	DAD<br>NACRS	ICD10: F50.2, F50.3

© 2022 Hundemer GL et al. *JAMA Network Open.*

Eating disorder not otherwise specified	DAD NACRS	ICD10: F50.8, F50.9
**Electrolyte Abnormalities**		
Hypokalemia (≤3.5 mmol/L)	OLIS	2823-3 [POTASSIUM:SCNC:PT:SER/PLAS:QN]<br>39789-3 [POTASSIUM:SCNC:PT:BLDV:QN]<br>6298-4 [POTASSIUM:SCNC:PT:BLD:QN]
Hyperkalemia (≥5.5 mmol/L)	OLIS	2823-3 [POTASSIUM:SCNC:PT:SER/PLAS:QN]<br>39789-3 [POTASSIUM:SCNC:PT:BLDV:QN]<br>6298-4 [POTASSIUM:SCNC:PT:BLD:QN]
Hyponatremia (≤130 mmol/L)	OLIS	2951-2 [SODIUM:SCNC:PT:SER/PLAS:QN]
Hypernatremia (≥150 mmol/L)	OLIS	2951-2 [SODIUM:SCNC:PT:SER/PLAS:QN]
Hypomagnesemia (≤0.50 mmol/L)	OLIS	2601-3 [MAGNESIUM:SCNC:PT:SER/PLAS:QN]
Hypophosphatemia (≤0.80 mmol/L)	OLIS	14879-1 [PHOSPHATE:SCNC:PT:SER/PLAS:QN]<br>24519-1 [PHOSPHATE:SCNC:PT:BLD:QN]
Metabolic alkalosis (Serum bicarbonate ≥30 mmol/L)	OLIS	14627-4 [BICARBONATE:SCNC:PT:BLDV:QN]<br>1959-6 [Bicarbonate:SCnc:Pt:Bld:Qn]<br>1960-4 [BICARBONATE:SCNC:PT:BLDA:QN]<br>1961-2 [BICARBONATE:SCNC:PT:BLDC:QN]<br>1962-0 [Bicarbonate:SCnc:Pt:Plas:Qn]<br>1963-8 [Bicarbonate:SCnc:Pt:Ser:Qn]
Metabolic acidosis (Serum bicarbonate ≤22 mmol/L)	OLIS	14627-4 [BICARBONATE:SCNC:PT:BLDV:QN]<br>1959-6 [Bicarbonate:SCnc:Pt:Bld:Qn]<br>1960-4 [BICARBONATE:SCNC:PT:BLDA:QN]<br>1961-2 [BICARBONATE:SCNC:PT:BLDC:QN]<br>1962-0 [Bicarbonate:SCnc:Pt:Plas:Qn]<br>1963-8 [Bicarbonate:SCnc:Pt:Ser:Qn]
**Patient Characteristics**		
Age	RPDB	
Sex	RPDB	
Index year		
Income quintile	RPDB	
Rurality (rural vs. urban)	RPDB	
Number of hospitalizations in last 2 years	DAD	
Previous healthcare visit for mental health	DAD NACRS	

© 2022 Hundemer GL et al. *JAMA Network Open.*

Outpatient psychiatry visit	OHIP	
Asthma	DAD<br>OHIP	ICD10: J45, J46<br>OHIP diagnosis code: 493
Chronic kidney disease	DAD<br>OHIP	ICD10: E102, E112, E132, E142, I12, I13, N08, N18, N19<br>OHIP diagnosis code: 403 585
Chronic obstructive pulmonary disease	DAD<br>OHIP	ICD10: J41, J42, J43, J44<br>OHIP diagnosis code: 491, 492, 496
Congestive heart failure	DAD<br>OHIP<br>NACRS	ICD10: I50<br>OHIP diagnosis code: 428<br>OHIP fee code: Q050
Inflammatory bowel disease	DAD<br>NACRS<br>OHIP	ICD10: K50, K51<br>OHIP diagnosis code: 555, 556
Diabetes	DAD<br>OHIP	ICD10: E10, E11, E12, E13, E14<br>OHIP diagnosis code: 250<br>OHIP fee code: Q040, K029, K030, K045, K046
Myocardial infarction	DAD	ICD10: I21, I22
Liver disease	DAD<br>NACRS<br>OHIP	ICD10: B16, B17, B18, B19, I85, R17, R18, R160, R162, B942, Z225, E831, E830, K70, K713, K714, K715, K717, K721, K729, K73, K74, K753, K754, K758, K759, K76, K77<br>OHIP diagnosis code: 070 571 573<br>OHIP fee code: Z551, Z554
Depression	DAD<br>NACRS	ICD10: F32.x, F33.x, F34.1, (dxtype='M')
Anxiety	DAD<br>NACRS	2016/17 onward<br>ICD10: F06.4, F40, F41, F93.0-2, F94.0<br><br>Pre-2016/17<br>ICD10: F40, F41, F42, F43, F48.8, F48.9; F93.1, F93.2
Personality disorder	DAD<br>NACRS	ICD10: F07.0, F21, F60,F61, F62, F68, F69
Substance abuse	DAD<br>NACRS	ICD10: F10-19, F55

Abbreviations: DAD, Discharge Abstract Database; ICD10, International Statistical Classification of Diseases and Related Health Problems Version 10; IKN, ICES Key Number; NACRS, National Ambulatory Care Reporting System; OHIP,

© 2022 Hundemer GL et al. *JAMA Network Open.*

Ontario Health Insurance Plan; OLIS, Ontario Lab Information System; RPDB, Registered Persons Database; SDS, Same Day Surgery Database.

© 2022 Hundemer GL et al. *JAMA Network Open.*

eFigure 1: Crude and adjusted odds ratios for the association between severe electrolyte abnormalities and subsequent eating disorder diagnosis.

Severe Electrolyte Abnormality	Crude OR (95% CI)	Forest Plot	Adjusted OR (95% CI)	Forest Plot
**Hypokalemia**	11.34 (6.89-18.65)		7.20 (4.22-12.26)	
**Hyperkalemia**	2.42 (1.53-3.83)		2.17 (1.27-3.72)	
**Hyponatremia**	8.67 (4.85-15.50)		5.30 (2.82-9.95)	
**Hypophosphatemia**	4.01 (1.64-9.82)		1.84 (0.66-5.13)	

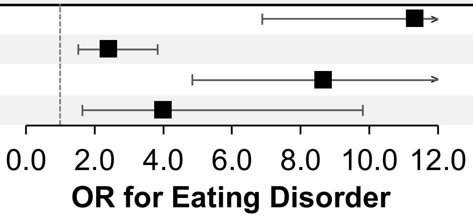

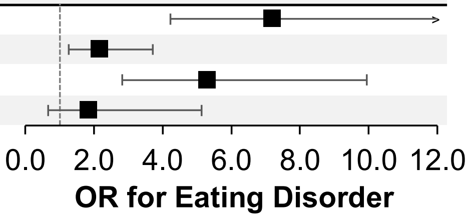

Severe electrolyte abnormalities were defined as follows: hypokalemia (serum potassium ≤3.0 mmol/L), hyperkalemia (serum potassium ≥6.0 mmol/L), hyponatremia (serum sodium ≤128 mmol/L), and hypophosphatemia (serum phosphate ≤0.60 mmol/L). The multivariable models adjusted for the following covariates: age, sex, year of index, income quintile, rural residence, number of hospitalizations in the prior two years, anxiety, asthma, chronic kidney disease, chronic obstructive pulmonary disease, congestive heart failure, depression, diabetes mellitus, inflammatory bowel disease, liver disease, myocardial infarction, personality disorder, and substance abuse.

Abbreviations: OR, odds ratio.

© 2022 Hundemer GL et al. *JAMA Network Open.*
